# Predictors for Physical Activity in Adolescent Girls Using Statistical Shrinkage Techniques for Hierarchical Longitudinal Mixed Effects Models

**DOI:** 10.1371/journal.pone.0125431

**Published:** 2015-04-30

**Authors:** Edward M. Grant, Deborah Rohm Young, Tong Tong Wu

**Affiliations:** 1 Department of Epidemiology and Biostatistics, School of Public Health, University of Maryland, College Park, MD, United States of America; 2 Department of Research & Evaluation, Kaiser Permanente Southern California, Pasadena, CA, United States of America; University of Rome, ITALY

## Abstract

We examined associations among longitudinal, multilevel variables and girls’ physical activity to determine the important predictors for physical activity change at different adolescent ages. The Trial of Activity for Adolescent Girls 2 study (Maryland) contributed participants from 8th (2009) to 11th grade (2011) (n=561). Questionnaires were used to obtain demographic, and psychosocial information (individual- and social-level variables); height, weight, and triceps skinfold to assess body composition; interviews and surveys for school-level data; and self-report for neighborhood-level variables. Moderate to vigorous physical activity minutes were assessed from accelerometers. A doubly regularized linear mixed effects model was used for the longitudinal multilevel data to identify the most important covariates for physical activity. Three fixed effects at the individual level and one random effect at the school level were chosen from an initial total of 66 variables, consisting of 47 fixed effects and 19 random effects variables, in additional to the time effect. Self-management strategies, perceived barriers, and social support from friends were the three selected fixed effects, and whether intramural or interscholastic programs were offered in middle school was the selected random effect. Psychosocial factors and friend support, plus a school’s physical activity environment, affect adolescent girl’s moderate to vigorous physical activity longitudinally.

## Introduction

The prevalence of childhood overweight and obesity has increased dramatically in the United States over the past decades such that 1/3 of adolescents are either overweight or obese [1]. Physical inactivity, a risk factor for obesity [2], is high. National data suggest that only 8 percent of adolescents participate in the recommended amount of physical activity; that is, 60 minutes per day [3]. The resulting health problems that can occur from physical inactivity and obesity, such as type 2 diabetes, high blood pressure, and sleep disorders, have been on the rise in children and adolescents in recent years [4]. The Council on Sports Medicine and Fitness and Council on School Health recommend that increasing physical activity in children and adolescents can be effective in reducing the prevalence of obesity and resulting future health problems [5].

Physical activity declines during adolescence. Kimm and colleagues observed a 64% decline among white and a 100% decline in black girls between the ages of 9–10 years to 18–19 years [6]. Further, the decline is steeper in girls than in boys [7]. In order to create effective interventions and strategies to increase physical activity among adolescent girls, it is useful to determine important predictors of physical activity among this population. While previous studies examined factors that influence physical activity participation in adolescents [8], most have used cross-sectional designs. In addition, while it is now recognized that physical activity is a complex behavior that is determined by factors at multiple levels [9], only a few have included variables at the individual, social, and environmental levels [10–13]. There are even fewer studies of multi-level predictors of longitudinal physical activity among adolescents [14].

Social ecological models support the notion that multi-level factors influence health behavior and are becoming increasingly used to determine predictors of physical activity [9]. The Trial of Activity for Adolescent Girls (TAAG) developed a social ecological framework that guided the development of the intervention and measurement protocols for the trial, which was designed to reduce the amount of physical activity decline among adolescent girls [15]. The model included psychosocial theories relevant to behavior change as well as appraisals of environmental settings in which adolescents can be active (e.g., schools, community, home). The theories used included operant conditioning (i.e., increased positive reinforcement, reduced barriers for physical activity and reduced positive reinforcement for sedentary behaviors) [16], social cognitive theory (i.e., increased self-efficacy, positive outcome expectations, positive role models) [17], and organizational change (i.e., enhancing environments to promote physical activity) [18]. Constructs from this framework can be used to predict physical activity longitudinally.

Using the TAAG social ecological framework, the trial’s subsequent study (Trial of Activity in Adolescent Girls 2 [TAAG 2]) provides longitudinal data for adolescent girls across six middle schools in Maryland to identify predictors of physical activity. The study used an objective measure of physical activity, accelerometry, which is considered the state of the art for physical activity assessment. The data are hierarchical and longitudinal in that there are individual level and school level predictors over two time points. It is necessary to use a mixed effects model in order to account for the within-individual correlations in observations over time, as well as to account for the within-group correlation in observations across the six schools. Young et al selected and estimated predictors of the TAAG at separate time points independently using the lasso variable selection technique [19]. In this paper, in order to determine significant predictors of physical activity over time, a new method of variable selection for linear mixed effects models was applied. In this model, not only the change of physical activity over time is considered, but also the predictors are allowed to be longitudinal (i.e., the values of predictors can change over time). Moreover, the clustering effects of schools can also be assessed in this model. The variable selection technique produces a parsimonious hierarchical longitudinal mixed effects model that selects important predictors of physical activity in adolescent girls over time and eliminates irrelevant predictors. These predictors can be used to guide future interventions.

## Methods

### Study Design

TAAG was a school and community-based, multisite trial targeted at girls with the goal of reducing the typical decline in physical activity during adolescence [20, 21]. The study was initially conducted at 36 middle schools across six geographically diverse areas in the United States, specifically Arizona, California, Louisiana, Maryland, Minnesota, and South Carolina. This study was approved by the University of Maryland Institutional Review Board.

As a part of TAAG, data were collected to assess the sustainability of the program in the spring of 2006 in a group of 8th grade girls at all 36 schools. A follow-up study was conducted in the spring of 2009 with the girls at the Maryland field center when the girls were in 11th grade. The data for this analysis include those collected during the 2006 and 2009 time points from the Maryland site.

### Data

Data for this analysis include those collected at the individual and school levels and are consistent with the constructs of the TAAG social ecological framework and its embedded behavior theories. Table 1 displays the key concepts used to identify relevant variables and the theories/constructs they represent. Girls completed surveys at 8^th^ and 11^th^ grade that queried on demographic, psychosocial, and perceived environmental factors known to be associated with adolescent MVPA [8]. Middle school-level variables were collected when the girls were in 8^th^ grade.

**Table 1:** Description of how key constructs apply to the settings and theories included in the Trial of Activity for Adolescent Girls Social Ecological Framework.

Characteristic	Environmental Setting	Operant conditioning	Social Cognitive theory	Organizational Change
Family sociodemographics	X			
Middle school demographics	X			
Other health behaviors		X		
Previous physical activity			X	
Physical activity self-efficacy			X	
Self-management strategies		X	X	
Physical activity enjoyment		X	X	
Physical activity barriers		X	X	
Outcome expectancies		X	X	
Physical activity social support			X	
Neighborhood amenities to facilitate physical activity	X			
School physical education requirements	X	X	X	X
School programs and policies supporting physical activity	X	X	X	X

### Individual Demographic and Health Behavior Factors

Girls self-identified as non-Hispanic white, black or African American, Hispanic or Latino, Asian/Pacific Islander, American Indian or Alaska Native, or other. They reported participation in free or reduced-price school lunch program, parent employment status, parent education, 1 or 2-parent household status, time spent at home alone unsupervised, and cigarette use. Height and weight were measured using standard procedures; body mass index (kg/m^2^) was calculated and girls were placed into normal weight, overweight, and obese categories based on the Centers for Disease Control and Prevention charts [22]. Percent fat was assessed from skinfold thicknesses using previously-described procedures [23].

Physical activity self-efficacy was assessed using an eight-item questionnaire developed for adolescent girls [24][25], with Cronbach alpha between 0.81 and 0.84 and test-retest correlation of 0.69 at 8^th^ grade [26]. Perceived physical activity barriers were assessed from an instrument adapted for TAAG with high internal consistency and reliability (Cronbach alpha = 0.88, 2-week test-retest reliability = 0.90) [27]. Outcome-expectancy value about physical activity was measured from nine items consisting of belief and corresponding value statements [28]. The belief statements had Cronbach alpha that ranged between 0.82 and 0.84 and the 8^th^ grade test-retest correlation was 0.68 [26], The Cronbach’s alpha for the value statements were between 0.92 and 0.94 and had a test-retest correlation of 0.58 [26], Seven items from the Physical Activity Enjoyment Scale (PACES) was used to determine enjoyment [29], and 1 item with a 5-point likert-type scale was used to identify enjoyment of physical education class [30]. PACES has been shown to be positively associated with physical activity among adolescent girls [29]. Depressive symptoms, which is associated with lower physical activity [31], was assessed from the Center for Epidemiological Studies-Depression Scale [32], a screen for major depressive disorder in adolescent populations [33]. Self-management strategies score was measured from an eight-item scale representing cognitive and behavioral strategies associated with physical activity, an instrument shown to explain small, but statistically significant, improvements in college-aged women’s physical activity [34].

Participation in sports, physical activity classes or lessons over the past year was assessed by asking if girls had participated in any of a list of 14 sports either at school or outside of school (e.g., softball, soccer, gymnastics) or any of a list of 17 classes or lessons (e.g., dance, martial arts, lacrosse). Items were summed. The number of years in which girls enrolled in physical education class was assessed for middle and high school.

### Social factors

Social support from parents and peers was assessed from separate scales developed for the Amherst Health and Activity Study [35], with Cronbach alpha from 0.74 to 0.79 and test-retest reliability of 0.86 [26]. Support from teachers was determined from two items regarding girls’ perception of how the teachers thought how important physical activity was for girls. A similar three-item scale was used to assess how boys perceived attitudes and a one-item scale for girls’ perceived attitudes [36]. Hours spent home alone after school without adult supervision, associated with 6^th^ grade physical activity, was assessed from two questions [37].

### Perceived environmental factors

Ten items were used to assess perceived neighborhood walkability and safety, with five response options ranging from disagree a lot to agree a lot [38]. Three items with four response options queried on the level of difficulty to get home from school every day from after school activities at school or to get to and get home from after school activities at another location. Test-retest reliability for the instrument, indicated by kappa coefficients, ranged from 0.38 to 0.41 [26]. Perceived access to a list of 14 physical activity facilities was queried with the following question: “Is it easy to get to and from this place from home or school” [39]? The intraclass correlation was 0.78 and Cronbach alpha ranged between 0.80 and 0.81 [26].

### Middle school factors

A 43-item, 30–45 minute structured interview was administered to school principals, which included questions on physical education policies, physical activity promotions, policies that support or constrain physical activity, transportation policies for active commuting, structured and unstructured physical activity opportunities, and collaborations with community organizations that provide physical activity programs. School websites were used to obtain each middle school’s race/ethnic, free and reduced price lunch, and mathematics and English state examination score profiles. MVPA in physical education class was assessed by the System for Observing Fitness Instruction Time [40].

### Outcome—MVPA

The outcome variable was the average minutes of average minutes of MVPA per day in the adolescent girls at each time point, which was collected using Actigraph accelorometers (model 7164, Fort Walton Beach, FL). Girls wore the accelerometers for 7 consecutive days over their right hip; data were reduced and processed using previously described methods [41]. Occasional missing data were replaced via an imputation technique based on the Expectation Maximum algorithm; details on the imputation method have been published [42]. MVPA was defined as 30-second accelerometer counts exceeding 1,499 [41]. Only girls with observations at both 2006 and 2009 time points were included in the analysis.

### Analyses

Missing data from the individual level predictors were imputed using the Sequential Regression Imputation Method [43].

### Modeling strategies

In order to account for the clustering effects of the school and the correlation between individual girls’ time points, a hierarchical longitudinal linear mixed effects (LME) model was used [44]. The fixed effects account for the mean responses of the model at different time points, which are shared across all participants. The random effects account for individual-specific (longitudinal) and cluster-specific (school-level) correlation. Ignoring the correlation within individuals and clusters observations can lead to incorrect conclusions, such as inaccurate standard error estimates in fixed effects and false hypothesis results [45].

There were a large number of variables collected at both time points that reflected the TAAG social ecological model (total of 66 variables of interest consisting of 47 fixed effects at the individual level, collected at both time points, and 19 school-level random effects predictors, collected only at 2006). While these variables represent the TAAG model and empirical studies indicate they are important predictors of adolescent physical activity, including all of these variables would lead a large model that is difficult to interpret and can lead to instability in estimating parameters. Additionally, as more variables are included in the model, the risk of correlation between variables, or multicollinearity, increases.

In order to reduce the size of the model for predicting MVPA, the doubly regularized selection and estimation technique for LME models developed by Wang et al. was applied to the fixed and random effects [46]. This method places two penalty functions on the LME model: A penalty for the fixed effects and a penalty for the random effects. This shrinks unimportant predictors to zero while selecting important predictors to be included in the model.

The process is summarized as: (1) the data are standardized such that each variable has mean 0 and norm 1; (2) a grid search of two tuning parameters associated with each penalty function is applied to the model (one for the fixed effects and one for the random effects), which control the strength of the shrinkage; (3) the model that gives a minimum Bayesian Information Criteria [47] is chosen as the optimal model that gives the best balance of fit and parsimony; and (4) the model is refit with only the selected fixed and random effects, with no constraints applied to the coefficients, using PROC GLIMMIX in SAS (SAS Institute, Carey, NC). Using this selection and estimation algorithm is more efficient than traditional methods—for example, best subset selection—where a model has to be fit for every possible combination of fixed and random effects and it is sometimes impossible to perform due to the huge number of possible combinations.

## Results

Demographic and descriptive statistics for the initial variables that were evaluated across the six schools are displayed in Table 1. The average daily MVPA across the 561 girls in 8^th^ grade was 20.8 minutes and was 19.9 minutes in 11^th^ grade. The average body mass index increased in five out of the six schools although the mean percent of those overweight and obese declined. Also of note is the reduction in difficulty getting to and from after school activities, the increase in hours spent home alone each week (mean (SD) 7.3 (7.8) hours in 8^th^ grade to 10 (8.9) hours in 11^th^ grade), and the decline in the number of sports team participation and participation in physical activity classes or lessons. Mean scores for the psychosocial and perceived neighborhood variables did not substantially change over time.

Also provided in Table 1 are the middle school level variables. There was substantial diversity in school level demographics (percent students who were non-Hispanic white ranged from 23 to 67), participation in free or reduced-price lunch (range 17% to 60%), and percent of students receiving passing scores on the state mathematics examination (range 28% to 76%). Less than 5% of students walked or bicycled to any of the middle schools. All but one school allowed unstructured physical activity opportunities either before, during, or after school. The number of school physical activity programs ranged from 7 to 20.

The variable selection analyses resulted in three selected fixed effects variables and one random effect variable. The fixed effects variables were self-management strategies, perceived barriers, and social support from friends. Although it was not selected, time was included in the final model to determine the effect of time on average daily MVPA. For the random effects variables, the predictor for offering interscholastic or intramural physical activity programs was selected. Therefore, the final LME model used for analysis contained four fixed effects and one random effect. All other 61 variables are eliminated from the pool of candidate variables.

As displayed in Table 2, self-management strategies (β = 0.13) and encouragement from friends (β = 0.42) were positively associated with daily MVPA, while perceived barriers was negatively associated with MVPA (β = -0.20). Time had a negative, but non-significant, association with daily MVPA. (See Table 3).

**Table 2:** Descriptive Characteristics for the Trial of Activity for Adolescent Girls (TAAG) (2006 8^th^ Grade) and TAAG2 (2009 11^th^ Grade) Studies, Maryland Field Site Cohort.

Characteristic	School 1 (n = 84)	School 2 (n = 102)	School 3 (n = 100)	School 4 (n = 79)	School 5 (n = 97)	School 6 (n = 99)	Total (n = 561)
Demographics							
White, n (%)	55 (65)	34 (33)	70 (70)	17 (22)	67 (69)	25 (25)	268 (48)
African American, n (%)	7 (8)	23 (23)	19 (19)	40 (51)	5 (5)	23 (23)	117 (21)
Hispanic, n (%)	7 (8)	19 (19)	7 (7)	9 (11)	5 (5)	27 (27)	74 (13)
Other race/ethnicity, n (%)	15 (18)	26 (25)	4 (4)	13 (16)	20 (21)	24 (24)	102 (18)
Grade began attending current school^a^							
8th Grade	6.1 (0.4)	6.1 (0.4)	6.1 (0.4)	6.4 (0.7)	6.1 (0.4)	6.3 (0.6)	6.2 (0.5)
11th Grade	9.01 (0.1)	9.2 (0.6)	9.1 (0.4)	9.2 (0.5)	9.0 (0.2)	9.1 (0.3)	9.1 (0.4)
Participation in the free or reduced price lunch program, n (%)							
8th Grade	27 (32)	29 (28)	14 (14)	43 (54)	7 (7)	26 (26)	146 (26)
11th Grade	20 (24)	27 (26)	12 (12)	35 (44)	7 (7)	20 (20)	121 (22)
Father employed, n (%)							
8th Grade	60 (71)	83 (81)	81 (81)	63 (80)	82 (85)	77 (78)	446 (80)
11th Grade	67 (80)	85 (83)	81 (81)	53 (67)	82 (85)	74 (75)	442 (79)
Mother employed, n (%)							
8th Grade	56 (67)	60 (59)	63 (63)	53 (67)	59 (61)	72 (73)	363 (65)
11th Grade	61 (73)	65 (64)	73 (73)	58 (73)	64 (66)	73 (74)	394 (70)
At least one parent attended college, n (%)							
8th Grade	26 (31)	70 (69)	64 (64)	21 (27)	75 (77)	53 (54)	309 (55)
11th Grade	30 (36)	71 (70)	61 (61)	17 (22)	72 (85)	51 (52)	302 (54)
Living with two parents, n (%)							
8th Grade	61 (73)	81 (79)	73 (73)	48 (61)	83 (86)	71 (72)	417 (74)
11th Grade	58 (69)	75 (74)	64 (64)	41 (52)	77 (79)	64 (65)	379 (68)
Cigarette use at least one time, n (%)							
8th Grade	25 (29.8)	7 (7)	5 (5)	11 (14)	1 (1)	12 (12)	61 (11)
11th Grade	51 (61)	86 (84)	83 (83)	63 (80)	76 (78)	74 (75)	433 (77)
Body mass index, kg/m^2^							
8th Grade	24.3 (5.8)	21.2 (4.1)	22.5 (5.5)	22.4 (5.1)	21.7 (4.6)	22.8 (5.3)	22.4 (5.1)
11th Grade	25.4 (6.0)	23.2 (4.4)	24.3 (6.1)	23.5 (5.4)	23.1 (5.1)	24.1 (5.1)	23.9 (5.4)
Overweight^b^ n (%)							
8th Grade	40 (48)	23 (23)	33 (33)	27 (34)	24 (25)	38 (38)	185 (33)
11th Grade	37 (44)	21 (21)	28 (28)	25 (32)	22 (23)	32 (32)	165 (29)
Obese^c^ n (%)							
8th Grade	25 (30)	8 (8)	16 (16)	16 (20)	12 (12)	18 (18)	95 (17)
11th Grade	23 (27)	8 (8)	14 (16)	11 (14)	11 (11)	12 (18)	79 (14)
Percent Fat							
8th Grade	34.0 (9.9)	29.1 (7.9)	31.0 (8.9)	28.9 (9.2)	30.7 (8.8)	31.1 (8.4)	30.8 (8.9)
11th Grade	33.4 (7.1)	31.1 (6.0)	31.6 (6.7)	28.4 (7.1)	31.1 (7.1)	30.2 (6.5)	31.1 (7.0)
Physical Activity							
Daily minutes of moderate to vigorous physical activity							
8th Grade	23.7 (12.9)	20.0 (9.7)	18.6 (9.4)	20.6 (9.7)	23.7 (13.1)	18.7 (7.9)	20.8 (10.7)
11th Grade	17.6 (9.1)	23.5 (13.9)	19.6 (11.6)	18.1 (11.1)	19.1 (10.4)	21.0 (10.9)	19.9 (11.5)
Currently taking a physical education class, n (%)							
8th Grade	82 (97)	86 (84)	99 (99)	69 (87)	94 (97)	74 (75)	504 (90)
11th Grade	75 (89)	81 (79)	94 (94)	72 (91)	95 (98)	80 (81)	497 (89)
Numbers of school sports team played on during the past year							
8th Grade	1.8 (2.0)	2.0 (1.8)	2.1 (2.3)	1.9 (1.8)	2.5 (1.9)	2.0 (1.9)	2.1 (2.0)
11th Grade	0.6 (1.0)	0.6 (0.9)	0.9 (1.0)	0.6 (0.9)	0.5 (0.8)	0.8 (1.3)	0.7 (1.0)
Number of outside school sports team played on during the past year							
8th Grade	2.2 (1.7)	2.0 (1.6)	2.4 (2.0)	2.5 (1.9)	2.9 (1.8)	2.0(1.6)	2.3 (1.8)
11th Grade	0.7 (1.2)	0.6 (0.8)	0.7 (1.6)	0.7 (1.4)	1.1 (1.5)	0.7 (0.8)	0.7 (1.2)
Number of physical activity classes or lessons taken during the past year							
8th Grade	2.2 (1.7)	2.3 (1.8)	2.7 (2.4)	2.5 (2.1)	2.7 (1.8)	1.9 (2.0)	2.4 (2.0)
11th Grade	0.5 (0.8)	0.6 (0.8)	0.9 (1.4)	0.8 (1.2)	0.9 (1.1)	0.8 (1.0)	0.8 (1.1)
Number of physical education classes taken in school							
8th Grade	1.5 (0.7)	1.6 (0.7)	1.5 (0.6)	1.6 (0.8)	1.5 (0.7)	1.5 (0.7)	1.5 (0.7)
11th Grade	0.6 (0.7)	0.4 (0.6)	0.3 (0.6)	0.4 (0.6)	0.6 (0.7)	0.4 (0.6)	0.4 (0.6)
Psychosocial							
Physical activity self-efficacy score							
8th Grade	38.8 (6.3)	26.7 (6.3)	30.7 (6.0)	28.6 (7.1)	28.8 (5.9)	27.7 (6.4)	28.5 (6.4)
11th Grade	28.9 (6.3)	27.1 (6.6)	29.0 (5.8)	29.6 (6.9)	28.2 (6.4)	28.1 (5.8)	28.4 (6.3)
Self-management strategies score							
8th Grade	25.4 (5.4)	26.9 (5.9)	27.9 (5.9)	25.1 (6.9)	27.5 (5.9)	25.9 (5.7)	26.5 (6.0)
11th Grade	26.1 (5.2)	27.2 (5.6)	27.4 (5.3)	26.1 (7.5)	26.8 (5.8)	27.0 (5.8)	26.8 (5.9)
Physical activity enjoyment score							
8th Grade	29.1 (6.7)	29.3 (5.0)	29.6 (5.0)	28.5 (6.8)	29.5 (5.3)	28.3 (6.4)	29.1 (5.9)
11th Grade	28.3 (6.7)	28.8 (4.8)	28.6 (5.3)	28.6 (6.4)	27.6 (6.3)	29.0 (5.5)	28.5 (5.8)
Physical activity barriers score							
8th Grade	22.6 (6.7)	21.9 (5.4)	20.3 (5.5)	21.6 (6.0)	21.5 (6.1)	21.6 (5.7)	21.6 (5.9)
11th Grade	22.2 (6.7)	23.1 (5.5)	20.9 (6.3)	21.2 (6.5)	22.1 (5.3)	21.6 (5.6)	21.9 (6.1)
Outcome expectancies (sum score)							
8th Grade	36.2 (6.2)	36.3 (5.0)	37.6 (5.1)	36.7 (6.3)	37.0 (4.9)	36.2 (6.0)	36.7 (5.6)
11th Grade	36.2 (5.9)	36.3 (4.5)	35.7 (4.7)	36.1 (6.0)	35.8 (5.0)	36.9 (4.7)	36.2 (5.1)
Outcome expectancy (value score)							
8th Grade	154.4 (50.2)	160.1 (39.3)	166.4 (40.9)	156.5 (49.9)	162.5(40.1)	157.6 (43.4)	159.8 (43.8)
11th Grade	140.0 (54.7)	154.3 (39.7)	144.2 (45.5)	149.0 (53.4)	146.5(43.4)	155.7 (48.7)	148.6 (47.5)
Physical education enjoyment score							
8th Grade	3.3 (1.4)	3.8 (1.2)	3.8 (1.1)	3.5 (1.3)	4.4 (1.3)	3.9 (1.2)	3.6 (1.3)
11th Grade	3.8 (1.4)	3.7 (1.3)	3.5 (1.2)	3.5 (1.4)	3.2 (1.5)	3.7 (1.3)	3.6 (1.3)
Depressive symptoms score							
8th Grade	19.1 (11.4)	13.8 (7.9)	14.4 (8.0)	17.7 (9.7)	13.9 (8.5)	16.3 (10.5)	15.7 (9.5)
11th Grade	17.4 (9.8)	13.9 (7.8)	15.5 (10.0)	16.4 (10.2)	13.7 (8.6)	15.7 (7.8)	15.4 (9.1)
Social							
Provides social support to others score							
8th Grade	2.8 (1.2)	2.5 (1.0)	2.8 (1.0)	2.6 (1.3)	2.7 (1.1)	2.5 (1.2)	2.6 (1.1)
11th Grade	2.6 (1.1)	2.3 (1.1)	2.6 (1.1)	2.6 (1.3)	2.3 (1.0)	2.5 (1.1)	2.4 (1.1)
Social support from friends score							
8th Grade	9.0 (2.7)	8.0 (2.7)	8.9 (3.0)	8.6 (3.7)	8.4 (3.2)	8.1 (2.8)	8.5 (3.0)
11th Grade	8.4 (2.9)	7.2 (3.1)	8.1 (3.0)	7.8 (3.5)	7.7 (3.0)	8.1 (2.9)	7.9 (3.1)
Social support from family score							
8th Grade	15.5 (5.4)	14.5 (5.0)	17.0 (4.2)	14.9 (5.4)	16.1 (4.9)	15.0 (4.8)	15.5 (5.0)
11th Grade	14.2 (4.9)	12.3 (5.6)	14.7 (4.8)	13.8 (5.4)	13.7 (5.0)	13.6 (4.4)	13.9 (4.8)
Positive support from physical activity from teachers score							
8th Grade	7.0 (2.5)	7.6 (2.3)	7.3 (2.5)	7.6 (2.4)	6.8 (2.2)	7.6 (2.2)	7.3 (2.3)
11th Grade	7.3 (2.1)	7.1 (2.3)	7.3 (2.2)	7.7 (2.3)	6.5 (2.2)	7.2 (2.3)	7.2 (2.2)
Positive support from physical activity from boys score							
8th Grade	9.9 (3.2)	10.1 (3.3)	19.3 (3.4)	9.8 (3.2)	9.9 (3.0)	10.4 (3.0)	10.1 (3.2)
11th Grade	9.7 (3.3)	10.0 (3.0)	10.4 (3.0)	10.3 (3.0)	10.1 (2.7)	10.6 (2.9)	10.2 (3.0)
Positive support for physical activity from girls score							
8th Grade	2.9 (1.3)	3.0 (1.2)	2.6 (1.1)	2.5 (1.3)	2.7 (1.0)	3.0 (1.2)	2.8 (1.2)
11th Grade	3.5 (1.1)	3.5 (1.1)	3.5 (1.1)	3.2 (1.3)	3.4 (1.1)	3.4 (1.1)	3.4 (1.1)
Hours spent alone per week							
8th Grade	7.9 (8.1)	5.0 (6.5)	7.7 (7.7)	10.2 (9.2)	8.3 (6.8)	6.7 (7.8)	7.3 (7.8)
11th Grade	9.7 (8.9)	8.1 (9.4)	9.0 (8.8)	14.1 (9.8)	8.9 (7.9)	10.8 (8.9)	10.0 (8.9)
Perceived Neighborhood							
Difficulty getting to community activity score							
8th Grade	2.0 (0.8)	2.1 (0.8)	1.9 (0.7)	2.1 (0.8)	1.9 (0.7)	2.2 (0.8)	2.0 (0.8)
11th Grade	1.0 (0.8)	0.8 (0.6)	0.7 (0.8)	0.9 (0.8)	0.9 (0.7)	2.0 (0.7)	0.9 (0.7)
Difficulty getting home from a community activity score							
8th Grade	1.9 (0.7)	2.1 (0.8)	1.6 (0.7)	2.1 (1.0)	1.8 (0.7)	2.0 (0.8)	1.9 (0.8)
11th Grade	0.8 (0.8)	0.8 (0.6)	0.6 (0.7)	0.8 (0.8)	0.8 (0.8)	0.9 (0.7)	0.8 (0.7)
Places to go in walking distance of home score							
8th Grade	3.8 (1.3)	3.7 (1.2)	3.4 (1.4)	3.9 (1.4)	3.3 (1.6)	3.7 (1.3)	3.6 (1.4)
11th Grade	3.8 (1.2)	3.6 (1.2)	3.0 (1.5)	3.5 (1.5)	2.8 (1.5)	3.6 (1.2)	3.4 (1.4)
Sidewalks on neighborhood streets score							
8th Grade	4.3 (1.2)	3.8 (1.4)	4.0 (1.5)	4.0 (1.4)	3.3 (1.9)	3.9 (1.4)	3.9 (1.5)
11th Grade	4.4 (1.1)	3.9 (1.4)	3.9 (1.3)	4.1 (1.4)	3.3 (1.8)	3.7 (1.5)	3.9 (1.5)
Bike or walking trails in neighborhood score							
8th Grade	2.7 (1.6)	3.3 (1.5)	2.8 (1.5)	2.5 (1.7)	2.9 (1.7)	3.2 (1.5)	2.9 (1.6)
11th Grade	3.1 (1.4)	3.6 (1.4)	2.7 (1.4)	2.6 (1.6)	2.9 (1.7)	3.3 (1.5)	3.1 (1.6)
Safe to walk or jog in neighborhood score							
8th Grade	3.9 (1.2)	3.8 (1.2)	4.2 (1.0)	3.5 (1.3)	4.1 (1.2)	4.0 (1.2)	3.9 (1.2)
11th Grade	4.1 (1.1)	4.1 (1.1)	3.9 (1.3)	3.6 (1.3)	4.1 (1.2)	4.1 (1.1)	4.0 (1.2)
Walkers and bikers seen in neighborhood score							
8th Grade	3.9 (1.2)	3.8 (1.2)	4.1 (1.1)	4.0 (1.2)	4.0 (1.2)	3.9 (1.2)	4.0 (1.2)
11th Grade	4.1 (1.0)	4.1 (1.0)	4.2 (0.9)	3.7 (1.3)	4.1 (1.1)	4.0 (1.1)	4.0 (1.1)
Traffic impedes the ability to walk in neighborhood score							
8th Grade	2.0 (1.3)	1.9 (1.1)	1.9 (1.2)	2.1 (1.4)	2.0 (1.2)	1.7 (1.1)	1.9 (1.2)
11th Grade	1.9 (1.0)	1.9 (1.0)	2.0 (1.2)	1.9 (1.1)	1.9 (1.1)	1.8 (1.1)	1.9 (1.1)
Crime in neighborhood score							
8th Grade	1.9 (1.1)	1.9 (1.2)	1.6 (1.0)	2.2 (1.3)	1.7 (1.1)	1.7 (1.1)	1.8 (1.1)
11th Grade	1.8 (1.2)	1.9 (1.3)	1.6 (1.0)	2.1 (1.2)	1.6 (0.9)	1.8 (1.0)	1.8 (1.1)
Children seen playing outdoors in neighborhood score							
8th Grade	3.8 (1.3)	3.5 (1.3)	3.7 (1.4)	3.9 (1.3)	3.6 (1.4)	3.5 (1.3)	3.7 (1.3)
11th Grade	3.5 (1.5)	3.8 (1.2)	3.6 (1.4)	3.5 (1.5)	3.6 (1.3)	3.7 (1.3)	3.6 (1.3)
Interesting things to view in neighborhood score							
8th Grade	3.2 (1.4)	3.3 (1.3)	3.2 (1.4)	3.2 (1.6)	3.3 (1.4)	3.0 (1.3)	3.2 (1.4)
11th Grade	3.1 (1.4)	3.4 (1.2)	3.0 (1.3)	2.8 (1.5)	3.1 (1.3)	3.3 (1.2)	3.1 (1.3)
Neighborhood streets are well-lit score							
8th Grade	3.2 (1.3)	3.3 (1.3)	3.5 (1.3)	3.0 (1.4)	3.0 (1.4)	3.3 (1.3)	3.2 (1.3)
11th Grade	3.5 (1.1)	3.5 (1.1)	3.5 (1.1)	3.2 (1.3)	3.4 (1.1)	3.4 (1.1)	3.4 (1.1)
Number of recreational facilities easy to get to from home or school							
8th Grade	6.2 (2.9)	7.2 (2.8)	7.3 (3.1)	5.7 (3.2)	7.3 (2.7)	6.9 (3.1)	6.8 (3.0)
11th Grade	7.6 (3.0)	8.7 (2.4)	9.2 (3.0)	6.5 (3.5)	8.4 (2.7)	8.3 (2.8)	8.2 (3.0)
Middle School							
Non-Hispanic white students in school, %	67	30	58	24	65	23	44.5 (19.0)
Students in school receiving free or reduced price lunch, %	31	44	24	60	17	41	35.5 (13.6)
Students in school passing state mathematics examination, %	53	61	73	28	76	48	56.5 (15.7)
Students in school passing state English examination, %	69	71	81	53	83	67	71.3 (9.6)
Average physical education class size in school	30	30	36	30	30	30	31.1 (2.3)
Weeks per year school physical education is required	36	27	36	39	32	27	32.5 (4.6)
Girls not meeting state requirements of 36 weeks per year, n, %	0	102	100	0	97	0	299 (53)
Girls whose schools had physical activity school events in the past year, n, %	84	102	0	0	0	99	285 (51)
Girls whose schools have interscholastic sports, n, %	84	0	100	79	97	99	459 (82)
Girls whose schools have intramural sports, n, %	84	0	100	79	97	99	459 (82)
Girls whose school had grounds changes in prior year, n, %	0	102	0	79	0	0	181 (32)
Girls whose school had policy changes encouraging physical activity in prior year, n, 5	0	0	0	0	97	0	97 (17)
Girls whose school had budget changes encouraging physical activity in prior year, n, %	0	102	0	0	0	99	201 (36)
Students who bike or walk to school, %	3	3	2	1	3	2	2.3 (0.7)
Girls whose school had unstructured physical activity opportunities before school, n, %	84	102	0	79	0	0	265 (47)
Girls whose school had unstructured physical activity opportunities during school, n, %	84	0	0	0	0	99	183 (33)
Girls whose school had unstructured physical activity opportunities after school, n, %	84	102	0	79	0	99	364 (65)
Physical activity programs in the school	14	20	8	7	9	16	12.5 (4.8)
Minutes in Moderate to vigorous physical activity during physical education class	5.9	16.2	6.2	6.2	6.7	13.7	9.4 (4.3)

^a^Data are expressed as mean (standard deviation) except where otherwise indicated.

^b^Overweight defined as between the 85^th^ and 94^th^ age and sex percentile growth charts.

^c^Obesity defined as equal or greater than the 95^th^ age and sex percentile growth charts.

**Table 3:** Regression Coefficient and Variance Estimates for Longitudinal Physical Activity using the Selected Fixed and Random Effects Variables.

	β or σ	95% Confidence Interval
Fixed Effects β (Individual Level)		
Time	0.51	(-1.6, 0.58)
Self-management strategies	0.12	(0.0002, 0.25)
Perceived barriers	-0.21	(-0.33, -.10)
Social support from friends	0.43	(0.19, 0.66)
Random Effects σ (School Level)		
Offer school interscholastic and intramural programs	3.51	n/a

Trial of Activity for Adolescent Girls, Maryland Field Site Cohort, 2006–2009.

Based on the nonzero variance components of the random effects selection, there was heterogeneity in MVPA across the population from the effects of interscholastic or intramural physical activity programs (σ = 6.64) offered in middle schools attended. This result can be interpreted as attending a middle school that offered interscholastic or intramural physical activity programs introduced additional school-level randomness to a girl’s MVPA.

## Discussion

This study was conducted to determine the longitudinal, multi-level predictors of physical activity among adolescent girls. Starting with 66 variables at the individual, social, perceived environment, and school levels that have been documented to be associated with physical activity, the doubly regularized selection and estimation technique reduced those to four variables to be included in hierarchical, longitudinal modeling. Our results indicated that greater use of self-management strategies, more social support from friends, fewer perceived barriers, and having interscholastic and intramural sports programs available in middle school predicted greater physical activity. These results provide useful information regarding the most salient individual, social, and school-level variables to be included in physical activity interventions for adolescent girls.

We identified multi-level factors that were longitudinally associated with physical activity. Hearst and colleagues examined predictors of 24-month change among boys and girls initially aged 10–16 years [14]. Among girls, they found that age, baseline MVPA, pubertal status, and perceived barriers—all individual level variables—were associated with follow-up physical activity. While our results regarding perceived barriers agreed with their results, Hearst et al did not find social support to be a significant factor nor did they measure physical activity-related cognitive/behavioral strategies.

The variables selected have tremendous potential for interventions and are, in fact, often included in youth physical activity interventions [48]. Many interventions are based in social cognitive theory, which posits that health behaviors are influenced by the environment (e.g., access to safe exercise settings and social support), the individual (e.g., beliefs about the causes and outcomes of behavior), and the behavior itself (e.g., perceptions about physical activity) [17] as well as the ecological model that includes sociocultural and environmental variables [49]. The selected variables represent these constructs. Potentially effective interventions for older adolescent girls should include developing cognitive and behavioral strategies, such as identifying benefits, reducing barriers, setting realistic goals, fostering social support (especially from friends) and building physical activity skills, and providing opportunities and programs that are easily accessible for girls.

It is interesting to examine the variables that were not selected as important predictors for longitudinal physical activity. Self-efficacy, or a person’s belief that she has the capability to successfully engage in a specific health behavior, is a key concept in the social cognitive theory [17]. However, it was not selected from the statistical shrinkage technique analysis. Hearst et al. also did not find self-efficacy to be associated with 24-month MVPA change [14]. Lytle et al. found that self-efficacy declined in the intervention compared with the control TAAG schools from 6^th^ to 8^th^ grade, although increased self-efficacy was an intervention mediator for increased MVPA [26]. They posited that girls exposed to the intervention may have become more aware of the challenges associated with increasing physical activity, which resulted in lower self-efficacy overall, albeit that those who were able to increase their self-efficacy became more active. Our previous cross-sectional work examining multi-level variables and MVPA found that in 8^th^ grade barriers, time spent alone, school-level socioeconomic status and academic achievement, and perceived neighborhood safety and in 11^th^ grade barriers, physical activity enjoyment, self-efficacy, availability of intramural programs at school, sidewalk availability, and close distance from home to any school were associated with MVPA [19]. Self-efficacy may be an inconsistent predictor of longitudinal physical activity across adolescence.

The change in MVPA from 8^th^ to 11^th^ grade was not significant. We previously found a significant decline in MVPA from 6^th^ to 8^th^ grade in metabolic equivalent-weighted MVPA but not for daily MVPA among the entire TAAG cohort [50]. From 6^th^ to 8^th^ grade the annual percent change in mean daily MVPA was -2.1% compared with -1.4% from 8^th^ to 11^th^ grade in the TAAG 2 cohort. While it is well-documented that physical activity declines during adolescence, there is less information available regarding the period in which it may stabilize—particularly when physical activity is assessed with accelerometry. Our results suggest that perhaps the slope of the decline becomes less steep throughout middle and high school for overall MVPA. More studies are needed that track physical activity throughout adolescence into young adulthood using accelerometry methods.

The variable selection technique used in the linear mixed effects model has great advantages and potential for clustered, longitudinal studies. It is an automatic and objective procedure to select the most important variables from a large pool of candidate variables. Although our initial selection of variables was theoretically based, no primary knowledge about which variables to be included is needed and it can be used as a great exploratory tool. Hearst et al. [14] and McKay et al. [13] chose several sets of predictor variables and fit models with different variable sets, which requires prior knowledge to determine which predictor variables to select. The employed regularization variable selection approach (by adding penalties to the objective function) considers all the variables simultaneously, not different combinations of variables as in traditional selection methods. Different than univariate selection in which correlation coefficients or univariate models are calculated/fit for each variable on the response and the variables with the highest ranks (e.g., according to p-values) get selected [51], the selection technique we used consider the correlation among variables so multicollinearity is avoided. Specifically, the method used in this paper is designed for linear mixed effects models so clustering and longitudinal effects can be considered. The item parceling used by Heitzler et al. [11] has potential problems as it depends on the order of variables and may also suffer from the subjective grouping of variables. Factor analysis, on the other hand, belongs to the “variable extraction” category of dimension reduction, instead of variable selection. In this approach, the original variables are projected into lower dimensional spaces by finding the “best” linear combinations of the original variables and those linear combinations are used as the new variables in subsequent modeling. In contrast, variable selection chooses a possible best subset from the original variables, allowing for meaningful interpretation of the results to be maintained.

Strengths of this study include a large, diverse sample of girls that were representative of middle school girls living in the Baltimore/Washington DC region in 2008, with excellent follow-up in 2011. Daily MVPA was measured by accelerometry and, for the most part, the self-reported measures had established validity and reliability. We were able to include a comprehensive set of variables that were multi-level and have been associated with MVPA. The variable selection technique that we used to build the hierarchical longitudinal model resulted in information useful for future intervention planning.

There are also study limitations. Only one geographical region was included in the study so results may not be generalizable to other areas of the U.S. We studied only girls, but since predictors of physical activity are known to differ between boys and girls [7], we would not expect the results to be generalizable to boys. There was only one follow-up point over a 3-year period and results may not be stable over a different follow-up.

The low amount of physical activity among adolescent girls is a significant public health problem and a critical factor for obesity prevention. We used a broad array of variables across multiple levels and applied an innovative statistical technique to identify the important predictors of longitudinal physical activity among middle to high school-aged girls. Greater use of self-management strategies, more social support from friends, fewer barriers, and having interscholastic and intramural sports programs available in middle school predicted greater MVPA. This is important information needed to create effective public health interventions and policies to halt physical activity decline and reverse the current child/adolescent obesity epidemic.
